# Expression of the phosphorylated MEK5 protein is associated with TNM staging of colorectal cancer

**DOI:** 10.1186/1471-2407-12-127

**Published:** 2012-03-30

**Authors:** Bang Hu, Donglin Ren, Dan Su, Hongcheng Lin, Zhenyu Xian, Xingyang Wan, Junxiao Zhang, Xinhui Fu, Li Jiang, Dechan Diao, Xinjuan Fan, Lei Wang, Jianping Wang

**Affiliations:** 1 The Sixth Affiliated Hospital, Sun Yat-sen University, Guangzhou, Guangdong 510655, China; 2 Shenzhen people's Hospital, Shenzhen 518035, China; 3 Guangdong Provincial Hospital of Traditional Chinese Medicine, Guangzhou, Guangdong 520120, China

## Abstract

**Background:**

Activation of MEK5 in many cancers is associated with carcinogenesis through aberrant cell proliferation. In this study, we determined the level of phosphorylated MEK5 (pMEK5) expression in human colorectal cancer (CRC) tissues and correlated it with clinicopathologic data.

**Methods:**

pMEK5 expression was examined by immunohistochemistry in a tissue microarray (TMA) containing 335 clinicopathologic characterized CRC cases and 80 cases of nontumor colorectal tissues. pMEK5 expression of 19 cases of primary CRC lesions and paired with normal mucosa was examined by Western blotting. The relationship between pMEK5 expression in CRC and clinicopathologic parameters, and the association of pMEK5 expression with CRC survival were analyzed respectively.

**Results:**

pMEK5 expression was significantly higher in CRC tissues (185 out of 335, 55.2%) than in normal tissues (6 out of 80, 7.5%; *P *< 0.001). Western blotting demonstrated that pMEK5 expression was upregulated in 12 of 19 CRC tissues (62.1%) compared to the corresponding adjacent nontumor colorectal tissues. Overexpression of pMEK5 in CRC tissues was significantly correlated to the depth of invasion (*P *= 0.001), lymph node metastasis (*P *< 0.001), distant metastasis (*P *< 0.001) and high preoperative CEA level (*P *< 0.001). Consistently, the pMEK5 level in CRC tissues was increased following stage progression of the disease (*P *< 0.001). Analysis of the survival curves showed a significantly worse 5-year disease-free (*P *= 0.002) and 5-year overall survival rate (*P *< 0.001) for patients whose tumors overexpressed pMEK5. However, in multivariate analysis, pMEK5 was not an independent prognostic factor for CRC (DFS: *P *= 0.139; OS: *P *= 0.071).

**Conclusions:**

pMEK5 expression is correlated with the staging of CRC and its expression might be helpful to the TNM staging system of CRC.

## Background

CRC is the third most commonly diagnosed cancer in males and the second in females, with over 1.2 million new cancer cases and 608700 deaths estimated to have occurred in 2008 worldwide [1]. In China, CRC remains the fourth most common malignant tumor, the fifth leading cause of cancer-related death and the incidence continues to increase [2]. At current rates, approximately 5%-6% of individuals will develop cancer of the colon or rectum within their lifetime [3]. The survival of CRC patients is directly associated with the tumor stage at the time of diagnosis. Patients with distant metastasis have a poor 5-year survival (12%), while patients with a localized disease have good prognosis (90%) [4]. However, currently, few markers besides TNM stage have been validated as diagnosis criteria in the world wild. Molecules involved in CRC progression might allow more accurate diagnosis the stage of CRC, which would improve efficacy of multimodal therapy and sparing patients from unnecessary procedures [5].

Mitogen-activated protein kinase kinases (MEKs/MAPKKs) represent a family of protein kinases upstream of MAP kinases that play a critical role in regulating cell proliferation and apoptosis. Mitogen/extracellular signal regulated kinase kinase-5 (MEK5) encodes a 444-amino-acid protein with an overall 40% homology to the other MEK proteins [6,7]. MEK5 is activated via the dual phosphorylation of its Serine 311 and Threonine 315 by MEKK2,3/Tpl2. Subsequently, activated MEK5 (pMEK5) specifically activates ERK5, and then the activated ERK5 phosphorylates substrates including MEF2, c-Fos, Fra 1, Sap-1, c-Myc and NF-*κ*B, most of which are oncogene [8]. Currently, the MEK5 has been reported as an important protein for sustaining tumor growth, most likely due to its supportive role in vasculogenesis and blood vessel homeostasis [9,10]. Moreover, MEK5 has been detected in several tumor cells or tissues, e.g. including prostate cancer [11-15], breast cancer [16-21], Hodgkin lymphoma [22,23] and malignant mesotheliomas [24]. The expression of MEK5 is high in these cancers and is an indicator of poor prognosis and/or induction of metastasis. However, the prognostic power has typically been based on total MEK5 expression and does not consider pMEK5.

In the present study, we have examined the expression levels of pMEK5 in CRC tissues using immunohistochemistry and Western blot. We explored possible correlations between pMEK5 expression and tumor progression, to determine its role during tumor development.

## Methods

### Patients and tissue specimens

This study was approved by the Institute Research Medical Ethics Committee of Sun Yat-Sen University. Written informed consent for using tissue samples was obtained from all patients. For this study, we performed an immunohistochemical assay of 335 paraffin-embedded samples of CRC and 80 adjacent normal mucosa tissue samples collected from patients in the Department of Gastrointestinal Surgery of the First Affiliated Hospital, Sun Yat-sen University (Guangzhou, P. R. China), between January 2000 and November 2006. Only patients who did not receive preoperative anticancer treatment were enrolled. Colorectal tissues were surgically removed from CRC patient, normal colorectal mucosa was obtained from the distal edge of the resection at least 5 cm from the tumor. Histomorphology of all tumor specimens and adjacent normal mucosa were confirmed with hematoxylin-eosin (H&E) staining. In according with the International Union against Cancer TNM classification system, we divided all CRC specimens into four groups. Clinical parameters such as sex, age at surgery differentiation grade, lymph node metastasis, distant metastasis, TNM stage, preoperative CEA level and preoperative CA19-9 level were collected. All specimens were fixed in 10% formalin and embedded in paraffin. Enrolled patients were followed for at least 5 years for survival analysis. After a median follow-up of 62.4 months, 102 (30.4%) of all patients had died. In addition, for Western blot analysis, we randomly selected 19 paired samples of CRC and corresponding adjacent normal tissues from patents that underwent surgical tissue resection at the Sixth Affiliated Hospital of Sun Yat-sen University (Guangzhou, P. R. China) during 2011. Written informed consent was obtained from each patient before surgery. All excised samples were obtained within 1 h after the operation to remove tumor tissue, and were then immediately kept in liquid nitrogen until further analysis.

### Tissue microarray and immunohistochemistry

After screening the H&E-stained slides for optimal tumor tissue and adjacent tissue at a distance of 5 cm from the tumor, we constructed TMA slides using the specimens from the 335 patients and the 80 specimens of primary CRC paired with normal mucosa in our Tissue Bank. For each specimen, two cores (1 mm diameter) were taken from histologically confirmed normal adjacent colorectal mucosa to construct the TMAs using Tissue Array (ALPHELYS, MINIPORE).

Immunohistochemistry was performed using the Envision System with diaminobenzidine (DAKO Cytomation, Glostrup, Denmark) according to the manufacture's protocol. In brief, antigen retrieval was performed with citrate buffer (pH 6.0) by heating in a microwave at a controlled final temperature of 121°C for 15 min. Sections were incubated overnight at 4°C with the primary antibody against pMEK5 (phosphor S311 + T315, Abcam, ab70608, diluted 1:800, HK, China), and then incubated with the secondary antibody (Envision System, Dako) for 30 min at room temperature. After rinsing with PBS three times each for 10 min, the sections were incubated with 3,3-diaminobenzidine (DAB) liquid for 1 min, counterstained with Mayer hematoxylin, dehydrated, and then mounted.

### Staining evaluation

For pMEK5 staining, tissue specimens were examined separately by two independent and experienced pathologists (D. Su and X. J. Fan) under double-blinded conditions without prior clinicopathologic data. Expression of pMEK5 in CRC was evaluated by scanning the entire tissue specimen under low magnification (×40) and then confirmed by high magnification (×400). An immunoreactivity score (IRS) system was applied. The staining intensity for pMEK5 was scored as: 0, negative (colorless); 1, weak (pallide-flavens); 2, moderate (yellow), and 3 strong (brown). The proportion of stained cells was also recorded as: 0 (none); 1 (1-25%); 2 (26-50%); 3 (51-75%); or 4 (76-100%). The intensity and extent scores were summed to yield a composite score of 0 to 7 for each specimen[25]. Composite scores of 0-4 were defined as indicating normal pMEK5 protein expression, and scores of 5-7 were considered to indicate pMEK5 overexpression.

### Western blot analysis

Nineteen pairs of randomly selected CRC patient tissue specimens and corresponding nontumor specimens were subjected to Western blot analysis. Total protein was extracted from frozen colorectal mucosa using the Whole Protein Extraction Kit (Fermentas, USA). Equal amounts of protein were separated by electrophoresis on a 12% SDS polyacrylamide gel and electrotransferred on a polyvinylidene difluoride (PVDF) membrane (Pall, Port Washington, New York, USA). The membrane was blocked with 5% Bovine Serum Albumin (BSA) for 1 h. The tissues were incubated with primary rabbit polyclonal antibodies against human pMEK5 (1:1000 dilution; Abcam, HK, China). The immunoreactive signals were detected with an enhanced chemiluminescence kit (Amersham Biosciences, Uppsala, Sweden). The procedures were conducted in accordance with the manufacturer's instructions. Levels of β-actin were used to normalize protein expression.

### Statistical analysis

All statistical analyses were carried out using the SPSS v.17.0 (SPSS, Inc., Chicago, IL). The Chi-square and Fishers' exact tests (used when an expected value in one of the cells was less than 5) for proportionality were used to analyze the relationship between the expression of pMEK5 and the clinicopathologic variables. The survival rates were calculated by the Kaplan-Meier method, and the differences between the survival curves were examined by the log-rank test. Univariate Cox proportional hazards regressions were applied to estimate the individual hazards ratio (HR) for the DFS and OS. The significant variables in the univariate analysis (*P *< 0.05) were then put into the multivariate analysis. The HR with 95% confidence interval was measured to estimate the hazard risk of individual factors. *P *values of less than 0.05 were considered statistically significant.

## Results

### pMEK5 expression in CRC tissue and normal colorectal mucosa samples

**Figure 1:**
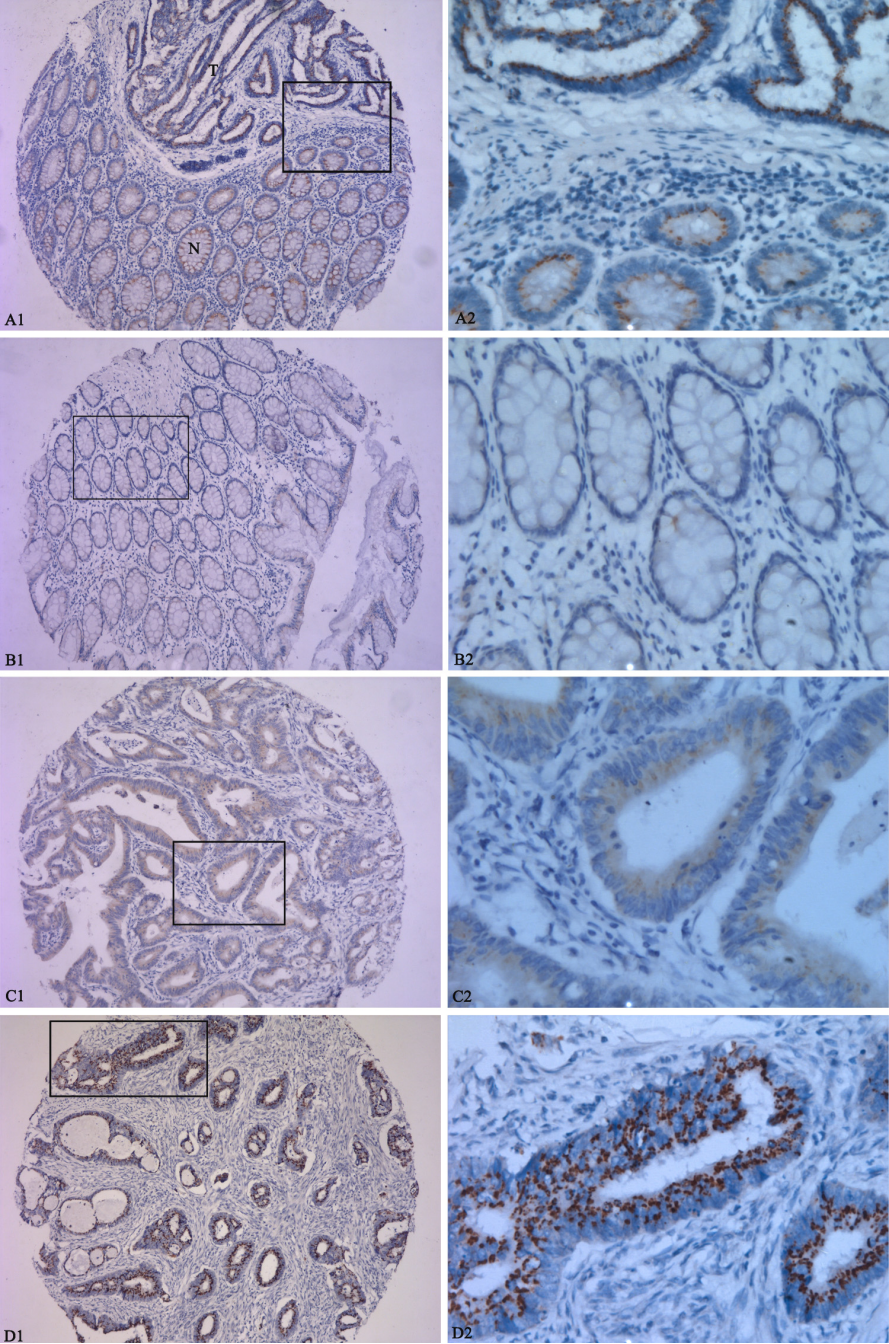
**Immunohistochemical analysis of the expression of pMEK5 protein**. pMEK5 expression was localized within the cytoplasm. A1 and A2, Elevated pMEK5 expression in the tumor cells of CRC tissue (T) compared with those of adjacent normal mucosa (N); B1 and B2, only weak staining of pMEK5 was detected in normal colorectal epithelial tissue; C1 and C2, moderately positive expression of pMEK5 in CRC without distant metastasis; D1 and D2, strongly positive expression of pMEK5 in CRC with distant metastasis (Left panels, ×100, right panels, ×400).

Immunostaining of pMEK5 in CRC tissues and normal mucosae was detected as brown-yellow granules in the cytoplasm (Figure 1). Overexpression of pMEK5 protein was observed in 186 of the 335 CRC tissues (55.5%; Figure 1D1/2). In contrast, only 6 of the 80 normal colorectal samples were upregulated for pMEK5, 74 samples had negative or weakly positive pMEK5 expression (Figure 1B1/2). Statistical analysis indicated that the CRC tissue samples exhibited significantly elevated expression of pMEK5 relative to that observed in the normal colorectal tissues (P < 0.0001; Figure 1A1/2; Table 1).

**Table 1:** pMEK5 expression in normal mucosa and CRC tissues

Tissue sample	All cases	pMEK5 protein		*P *value
			
		Normal expression	Overexpression	
Normal mucosa	80	74(92.5%)	6(7.5%)	< 0.001
Carcinomas	335	149 (44.5%)	186 (55.5%)	

**Figure 2:**
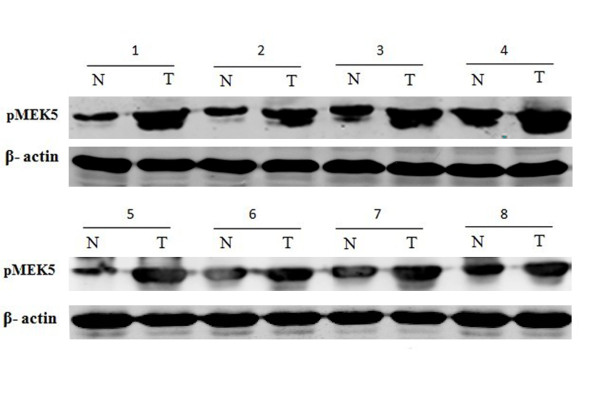
**Western blot analysis of pMEK5 protein expression**. Western blot analysis of pMEK5 proteins expressed in eight pairs represents colorectal tumor tissues (T) and their matched adjacent nontumor tissues (N). Expression level was normalized with β-actin.

To confirm the expression levels of pMEK5 seen by immunostaining in the specimens from our TMA, we examined the expression of pMEK5 protein by Western blot analysis in 19 randomly selected pairs of CRC tissues and their matched nontumor colorectal tissues. In 12 of 19 (62.1%) CRC patients, pMEK5 protein was upregulated in tumor tissues compared with their adjacent nontumor colorectal tissues (Figure 2).

### Correlation of pMEK5 protein expression and clinicopathologic parameters

The relationship between immunohistochemical pMEK5 expression in CRC tissues and various clinicopathologic characteristics is shown in Table 2. The expression (staining) of pMEK5 was highly correlated with depth of invasion (*P *= 0.001), lymph node metastasis (*P *< 0.001), distant metastasis (*P *< 0.001), preoperative CEA level (*P *< 0.001) and TNM stage (*P *< 0.001). However, pMEK5 expression was not significantly associated with age at surgery, sex, tumor location, differentiation grade or preoperative CA19-9 level (*P *> 0.05; Table 2).

**Table 2:** Correlation between pMEK5 expression and clinicopathologic characteristics

Variable	Cases	pMEK5 protein		*P *value
			
		Normal expression	Overexpression	
Sex				0.055
Female	158	79(50.0%)	79(50.0%)	
Male	177	70(39.5%)	107(60.5%)	
Age at surgery, years				0.835
< 65	189	85(45.0%)	104(55.0%)	
≥ 65	146	64(43.8%)	82(56.2%)	
Tumour location				0.440
Right colon	57	21(36.8%)	36(63.2%)	
Left colon	99	45(44.5%)	54(54.5%)	
Rectum	179	83(46.4%)	96(53.6%)	
pT (invasion depth)				0.001
T1+ T2	62	39(62.9%)	23(37.1%)	
T3+ T4	273	110(40.3%)	163(59.7%)	
pN (lymph node metastasis)				< 0.001
N0	203	113(55.7%)	90(44.3%)	
N1-N2	132	36(27.3%)	96(72.7%)	
pM (distant metastasis)				< 0.001
M0	305	146(47.9%)	159(52.1%)	
M1	30	3(10.0%)	27(90.0%)	
TNM stage				< 0.001
I	49	35(71.4%)	14(28.6%)	
II	145	78(53.8%)	67(46.2%)	
III	111	33(29.7%)	78(70.3%)	
IV	30	3(10.0%)	27(90.0%)	
Differentiation grade				0.074
Well	29	17(58.6%)	12(41.4%)	
Moderate	278	124(44.6%)	154(55.4%)	
Poorly	28	8(28.6%)	20(71.4%)	
Preoperative CEA level				< 0.001
< 5 ng/ml	190	102(53.7%)	88(46.3%)	
≥ 5 ng/ml	145	47(32.4%)	98(67.6%)	
Preoperative CA19-9 level				0.251
< 60 U/ml	296	135(45.6%)	161(54.4%)	
≥ 60 U/ml	39	14(35.9%)	25(64.1%)	

### Survival analysis

**Figure 3:**
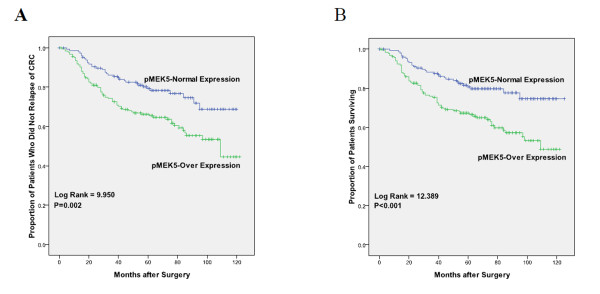
**Survival analysis of primary CRC patients (n = 335)**. Kaplan-Meier estimates of the DFS (A) and OS (B) according to pMEK5 expression in 335 patients. The DFS and OS were significantly lower in patients with pMEK5 overexpression when compared with patients who had normal pMEK5 expression. P values were calculated using the log-rank test.

The mean patient follow-up time was 71.5 months with a median value of 62.4 months. The 5-year OS rate of the 335 patients with primary colorectal cancer was 69.6%, with 102 deaths observed during the follow-up period. The 5-year DFS rate was 67.8%. During the time of follow-up, 82 patients (24.5%) developed distant metastasis or local recurrence. According to the univariate analyses, patients whose localized colorectal tumors overexpressed pMEK5 had a significantly lower 5-year DFS than patients with normal pMEK5 expression in their tumors (hazard ratio: 1.898; 95%CI: 1.264-2.850; *P *= 0.002; Figure 3A; Table 3). Similarly, the 5-year OS was also significantly lower in CRC patients with pMEK5 overexpression than CRC patients with normal pMEK5 expression in their tumors (HR: 2.107; 95%CI: 1.376-3.227; *P *< 0.001; Figure 3B; Table 4). In addition, tumor location (*P *= 0.018), lymph node metastasis (*P *< 0.001), distant metastasis (*P *< 0.001), TNM stage (*P *< 0.001), differentiation grade (*P *< 0.001), preoperative CEA level (*P *= 0.004) and preoperative CA19-9 level (*P *= 0.013) were statistically significantly associated with DFS and OS.

**Table 3:** Cox proportional hazards model univariate and multivariate analyses of individual parameters for correlations with disease-free survival (DFS)

Variable	Univariate analysis			Multivariate analysis		
	
	HR	CI(95%)	*P *value	HR	CI(95%)	*P *value
Sex						
Female	1					
Male	0.966	0.662-1.409	0.856			
Age at surgery, years						
< 65	1					
≥ 65	1.228	0.841-1.794	0.287			
Tumor location						
Colon	1			1		
Rectum	1.602	1.083-2.368	0.018	1.874	1.255-2.801	0.001
TNM stage						
I-II	1			1		
III-IV	2.851	1.933-4.204	< 0.001	2.383	1.572-3.614	< 0.001
pT (invasion depth)						
T1-T2	1					
T3-T4	1.398	0.822-2.379	0.216			
pN (lymph node metastasis)						
N0	1					
N1-N2	2.351	1.607-3.439	< 0.001			
pM (distant metastasis)						
M0	1					
M1	7.250	4.619-11.38	< 0.001			
Differentiation grade						
Well and Moderate	1			1		
Poorly	3.082	1.830-5.188	< 0.001	2.988	1.746-5.114	< 0.001
Preoperative CEA level						
< 5 ng/ml	1			1		
≥ 5 ng/ml	1.748	1.196-2.556	0.004	1.192	0.789-1.801	0.405
Preoperative CA19-9 level						
< 60 U/ml	1			1		
≥ 60 U/ml	1.926	1.146-3.236	0.013	1.779	1.035-3.056	0.037
Phospho-MEK5 protein						
Normal expression	1			1		
Overexpression	1.898	1.264-2.850	0.002	1.388	0.900-2.141	0.139

**Table 4:** Cox proportional hazards model univariate and multivariate analyses of individual parameters for correlations with overall survival (OS)

Variable	Univariate analysis			Multivariate analysis		
	
	HR	CI(95%)	*P *value	HR	CI(95%)	*P *value
Sex						
Female	1					
Male	1.008	0.683-1.488	0.967			
Age at surgery, years						
< 65	1					
≥ 65	1.201	0.813-1.773	0.357			
Tumor location						
Colon	1			1		
Rectum	1.560	1.044-2.331	0.030	1.794	1.191-2.701	0.005
TNM stage						
I-II	1			1		
III-IV	2.884	1.932-4.306	< 0.001	2.287	1.490-3.510	< 0.001
pT (invasion depth)						
T1-T2	1					
T3-T4	1.373	0.794-2.376	0.257			
pN (lymph node metastasis)						
N0	1					
N1-N2	2.346	1.586-3.469	< 0.001			
pM (distant metastasis)						
M0	1					
M1	7.292	4.601-11.558	< 0.001			
Differentiation grade						
Well and Moderate	1			1		
Poorly	3.206	1.900-5.409	< 0.001	2.853	1.658-4.907	< 0.001
Preoperative CEA level						
< 5 ng/ml	1			1		
≥ 5 ng/ml	1.904	1.287-2.817	0.001	1.280	0.834-1.964	0.259
Preoperative CA19-9 level						
< 60 U/ml	1			1		
≥ 60 U/ml	1.911	1.119-3.262	0.018	1.587	0.905-2.782	0.107
Phospho-MEK5 protein						
Normal expression	1			1		
Overexpression	2.107	1.376-3.227	< 0.001	1.518	0.964-2.388	0.071

Multivariate analysis was performed using the Cox proportional hazards model for all of the significant variables in the univariate analysis; however, T stage, lymph node metastasis, and distant metastasis were collinear with TNM stage, so we excluded lymph node metastasis and distant metastasis from the final model [26]. The results from the multivariate analyses for DFS and OS showed that the pMEK5 expression (*P *> 0.05) were not independent prognostic factors for overall survival (Table 4).

## Discussion

Malignant growth is often described as increased cellular proliferation induced by uncontrolled activation of mitogenic signaling pathways [27]. The MEK5/ERK5 signaling pathway regulates a wide variety of cellular processes during development and also plays an important role in human malignant diseases[28]. The MEK5/ERK5 signaling pathway is upregulated in many types of cancer, including prostate cancer [11-15], breast cancer [16-21], and oral squamous cell carcinoma [29]. In metastatic prostate cancer, strong MEK5 expression is correlated with bony metastases, and less favorable prognosis is caused by upregulated BMK1-induced activator protein-1 (AP-1) activity, a consequent induction of a high level of matrix metallo-protease-9 (MMP-9) and augmented invasive potential [11]. In oral squamous cell carcinoma, Sticht and colleagues [29] found that the high pERK5 expression in oral squamous cell carcinoma was associated with an advanced tumor stage and the presentation of lymph node metastases. In breast cancer cells, the activated oncogene STAT3 binds to the promoter regions of MEK5 and induces transcription, conferring a critical survival signal [17]. Moreover, during chemotherapeutic-induced apoptosis, overexpression of MEK5 in breast cancer cells provides a key survival signal for chemoresistence [30]. These findings suggested that pMEK5 might be involved in tumor progression and metastatic events.

Our original studies find that activation of MEK5 (pMEK5) was increased in CRC cases. We examined the expression of pMEK5 by immunohistochemistry and western blot, and found that pMEK5 protein expression was increased in CRC compared with adjacent normal mucosa from the same individual. Furthermore, we find that the pMEK5 expression status was significantly correlated with progression of CRC.

In this study, we examined the expression of pMEK5 in 335 cases of CRC by immunohistochemistry. pMEK5 was highly expressed with depth of invasion, especially in T3, T4 carcinomas, but not in T1 and T2 carcinomas (*P *= 0.001). Therefore, the depth of invasion is a very important prognostic factor, suggesting that pMEK5 can be used as a biomarker to identify subsets of CRC patients with more aggressive feature.

Another interesting finding was that lymph node metastases were detected more frequently in CRC patients with pMEK5 overexpression, when compared with normal cases of pMEK5 expression (*P *< 0.001). The same tendency was observed in distant metastases (*P *< 0.001), suggesting that the pMEK5 protein may play a role in tumor metastasis, which is consistent with previous studies of prostate cancer [11]. Consistently, we found that pMEK5 expression in CRC was positive correlation with TNM stage, and pMEK5-overexpression was significantly higher in cases at advanced stage than those at early stage.

One of the greatest challenges in colorectal cancer management is to accurately diagnose the tumor stage and determine proper adjuvant therapy. In early CRC, surgery may be all that is required [31,32]. In more advanced cancer, other treatments, such as chemotherapy or radiation therapy, may be required [33,34]. So, detection of the tumor stage seemed to be very important. Currently, the TMN staging system of tumors continues to be the gold standard for clinical stages in world wild. However, identifying the lymph node metastasis and distant metastasis with TNM staging system is difficult. For the TNM staging system, the screening of distant metastasis is primarily determined by imaging studies (CT, MRI or PET-CT), ultrasound, and by direct visualization and palpation during operation. These common examination methods have poor detection of early distant metastasis, such as CRC patients with less than 1 cm liver metastasis [35]. Our study found that pMEK5 could be helpful in the pathological study of CRC, especially for the classification of different stages in CRC and early distant metastasis, which would improve efficacy of multimodal therapy and allocation of resources, sparing patients from unnecessary procedures.

Moreover, Kaplan-Meier analysis of the survival curves showed a significantly worse 5-year disease-free (log-rank test, *P *= 0.002) and 5-year overall survival rate (log-rank test, *P *< 0.001) for patients whose tumors overexpressed pMEK5. This suggests that pMEK5 protein is a biomarker identifying a poor prognosis for patients with colorectal cancer. However, multivariate analysis showed that pMEK5 expression is not a biomarker for a worse prognosis (DFS: *P *= 0.139; OS: *P *= 0.071). Our results demonstrate that pMEK5 expression was correlated with a worse prognosis, but that pMEK5 was not an independent prognostic factor for CRC. The key reason may be due to the lack of adequate cases.

However, this study is retrospective look at the expression of pMEK5 in the CRC tissue, it is not able to establish if the expression of the pMEK5 is after CRC has occurred. And the mechanism of actions for pMEK5 protein is not known and needs further investigation. Patients with colorectal cancer whose pMEK5 expression is elevated may require a more powerful adjunctive therapy and intensive follow-up. Whether pMEK5 has value clinically as a biomarker for therapeutic approaches in patients with colorectal cancer should be followed up with additional appropriately, designed studies.

## Conclusions

In summary, we have provided original data to support an important role of pMEK5 overexpression in human CRC. Overexpression of the pMEK5 protein could be helpful in the classification of different stages in CRC, and it can be used as an adjunct to the TNM stage system to assess clinical stage.

## Competing interests

The authors declare that they have no competing interests.

## Authors' contributions

BH, DLR, LW and JPW designed and directed the study. BH, XHF, HCL, ZYX, DCD performed the majority of the experiments and composition of the manuscript. DS, JXZ, XYW, XJF and LJ were responsible for data collection and analysis, and reviewing and scoring the degree of immunostaining of sections. All authors have read and approved the final manuscript.

## Pre-publication history

The pre-publication history for this paper can be accessed here:

http://www.biomedcentral.com/1471-2407/12/127/prepub
